# Long-term activity of social insects responsible for the physical fertility of soils in the tropics

**DOI:** 10.1038/s41598-023-39654-w

**Published:** 2023-07-31

**Authors:** Ary Bruand, Adriana Reatto, Michel Brossard, Pascal Jouquet, Éder de Souza Martins

**Affiliations:** 1 Institut des Sciences de la Terre d’Orléans (ISTO), UMR7327, UO, CNRS, BRGM, Observatoire des Sciences de l’Univers en région Centre (OSUC), Université d’Orléans, 1A Rue de la Férollerie, 45071 Orléans, Cedex 2, France; 2 Secretaria de Pesquisa e Desenvolvimento, Empresa Brasileira de Pesquisa Agropecuária (Embrapa), Parque Estação Biológica-PqEB s/no, Brasília, DF Brazil; 3 Institut de Recherche pour le Développement (IRD), Eco&Sols, UMR IRD, INRAE, CIRAD, Institut Agro, University of Montpellier, Montpellier, France; 4 Institut de Recherche pour le Développement (IRD), Institute of Ecology and Environmental Sciences of Paris (iEES Paris), UPEC, CNRS, IRD, INRAE, UMR Sorbonne Université, Paris, France; 5 Empresa Brasileira de Pesquisa Agropecuária (Embrapa Cerrados), Brasília, DF Brasil

**Keywords:** Biogeochemistry, Environmental sciences

## Abstract

Ferralsols correspond to the red and yellow soils that are common in the tropics. They are deeply weathered but physical fertility is high because they exhibit a strong microgranular structure whose origin is still actively debated. In the present study, we looked for evidence of the biological origin of the structure resulting from soil fauna activity. We present results recorded with Brazilian Ferralsols developed under native vegetation. It was found that the Ferralsols studied exhibit morphological features related to the activity of social insects. We showed the presence of potassium 2:1 clays originating from the saprolite in the microaggregates of all the Ferralsols studied. These 2:1 clays were earlier discussed as markers of long-term termite activity. This highlights the threat that weighs on the physical fertility of these soils, and more broadly on the water cycle in the tropical regions concerned, if intensive agriculture reduces the soil fauna biodiversity, as indicated by several studies.

## Introduction

Most yellow or red soils found in the South American, African and Asian tropics are Ferralsols. They cover 750 million hectares worldwide^1^, which corresponds to about 5% of the total land surface area and 14% of the land surface area in the tropics. They result from a long sequence of deep weathering under conditions that in most cases have prevailed for several million years^1–5^. In these soils, easily weatherable primary minerals such as glasses and ferromagnesian minerals, and even the more resistant feldspar and micas have disappeared completely^6^. Consequently, their fine fraction is essentially made up of low-activity clay (mainly kaolinite) and of iron and aluminium sesquioxides^7–9^. As a result, Ferralsols are characterized by extremely low native chemical fertility, resulting from very low nutrient reserves, low pH, high phosphorus retention by oxide minerals, and low pH-dependent cation exchange capacity^6,10^. On the other hand, most Ferralsols are characterized by a well-developed microgranular structure^2,11–17^ and poor horizonation with very diffuse limits between horizons^1^. Thus, most Ferralsols exhibit high physical fertility resulting from high porosity, a high infiltration rate, high available water retention and low resistance to root penetration^6^.

The role of soil social insects, particularly termites, in the formation of their microgranular structure which is responsible for most of their physical fertility remains highly debated^2,11,19–23^. This is a major issue because if the long-term activity of soil social insects is responsible for the physical fertility of Ferralsols^23–27^, the development of agriculture following native vegetation clearing and its consequences on soil biodiversity^28–34^ can lead to the disappearance of the soil social insects responsible for the microstructure and its regeneration.

In South America, Ferralsols are widespread, most being located in the Brazilian Cerrado which is a savanna biome often regarded as a land reserve for agribusiness expansion^33,35^. The pressure on the land is particularly high, with 92 million hectares of native Cerrado vegetation already cleared by 2019 to make way for intensive agriculture^36^. In this context, the microgranular structure of the Ferralsols of the Cerrado biome which represent 280 million hectares has been the object of numerous studies^2,16–19,23,37^ but only a few concern the effects of native Cerrado vegetation clearing and the development of intensive agriculture on the soil biodiversity^24,38^. As both the microgranular structure and the communities of soil insects are particularly well developed in Ferralsols under native Cerrado vegetation, Ferralsols are highly favorable to the study of the processes responsible for the microgranular structure development and subsequently, for their native physical fertility.

Questions have been raised about the possible degradation of biodiversity in these soils following the development of intensive agriculture in this Brazilian region^30–33^, the sustainability of intensive agriculture on these soils^28–33^, the consequences for ecosystem services^39^ and climate changes at local and regional scales^35,40–42^.

The contribution of the long-term activity of the soil social insects to the formation of the microgranular structure of the Ferralsols under native vegetation is a current topic of debate^11,21^. Indeed, if it is established that it is the main process of the formation of this microgranular structure, it will then be urgent to study the consequences of the development of intensive agriculture on the soil macrofauna and its repercussions on the characteristics of the subsoil structure of Ferralsols and on the physical fertility of the soil in general.

## Results and discussion

In this study, we looked for evidence of the biological origin of the microgranular structure of Ferralsols that was related to long-term activity of social insects. To achieve this, we looked for the presence of biological structures related to termite or ant activity and for the presence of potassium 2:1 clays in the microgranular aggregates of the ferralic B horizons of Ferralsols^1^ selected under native forest vegetation in the Brazilian Cerrado biome and developed on a large range of parent materials. These 2:1 clays were indeed recently discussed as being markers of termite activity in Ferralsols^21^.

### Structures related to the activity of social insects

**Figure 1:**
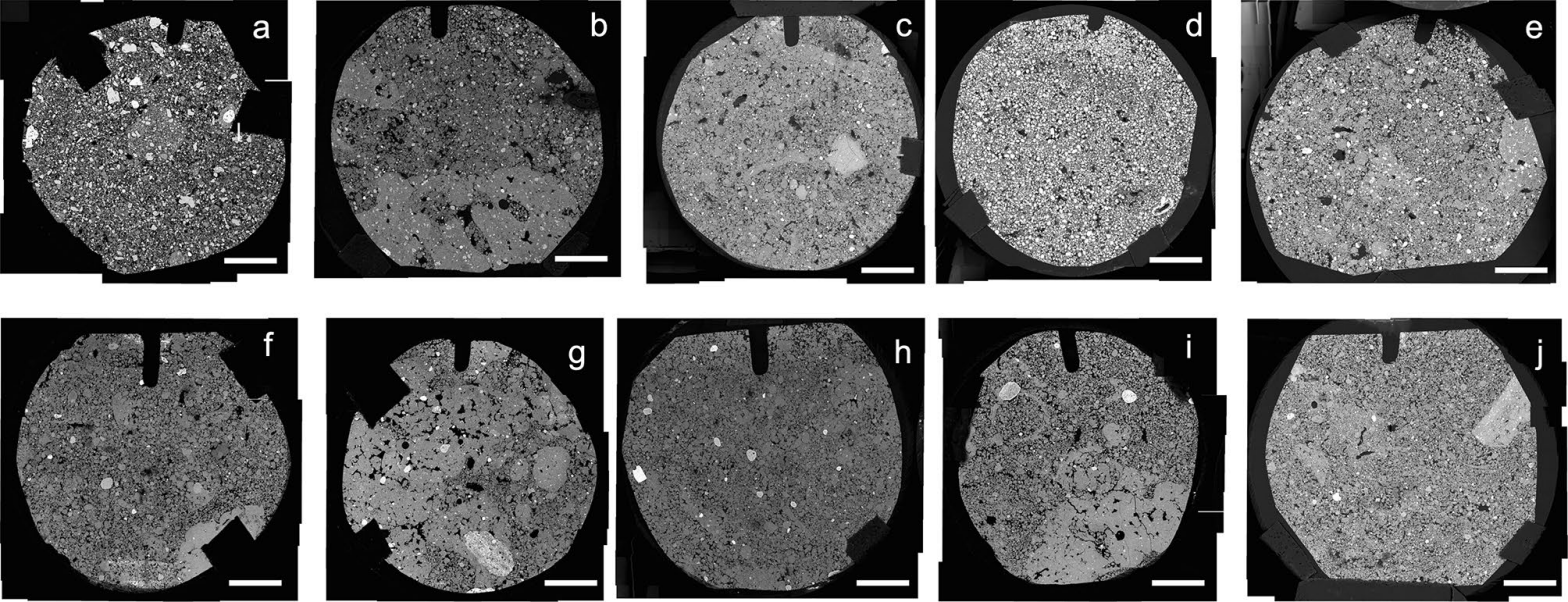
Backscattered electron scanning images (BESI) at low magnification of polished sections of BF1 (**a**), BF2 (**b**), BF3 (**c**), BF4 (**d**), BF5 (**e**), BF6 (**f**), BF7 (**g**), BF8 (**h**), BF9 (**i**), and BF10 (**j**) showing the relative distribution of areas with highly to moderately separated microaggregates forming a strong microgranular structure and areas with coalesced microaggregates forming a moderate to weak microgranular structure. Bar length: 5 mm.

Observation of the structure at low magnification showed the presence of two types of microgranular structure: areas with a strong microgranular structure with highly to moderately separated subangular microaggregates, and areas with a moderate to weak microgranular structure with coalesced subrounded microaggregates (Fig. 1). These two types of structures were present in all the ferralic B horizons studied but they were particularly easily recognizable in BF1 (Fig. 1b), BF3 (Fig. 1d), BF7 (Fig. 1g), BF9 (Fig. 1i) and BF10 (Fig. 1j). Their presence in ferralic B horizons was discussed very early in African Ferralsols^43^ and recently in Brazilian Ferralsols^23^ as being related to the bioturbation activity of termites.

Several ferralic B horizons among the ten horizons studied showed structures which result from soil macrofauna activity^18,23,43,44^. Thus, BF2 showed the presence in areas with highly coalesced microaggregates of cross-sections of several galleries or cavities, from 2 to 10 mm in size, partially filled with loosely-packed microaggregates and resulting from termite or ant activity (Fig. 1b, Supplementary Fig. 1). Then, BF7 showed the presence in an area with a strong microgranular structure of the cross-section of a gallery or cavity, 5 mm in size and fully filled with loosely-packed microaggregates, resulting from termite activity as indicated by the walls which result from microaggregates glued together thus forming a close packing (Fig. 1g, Supplementary Fig. 2). In BF9, the cross-section of a gallery or cavity resulting from termite or ant activity was observed in an area with coalesced microaggregates, from 3 to 6 mm in size and fully filled with loosely or closely-packed microaggregates (Fig. 1i, Supplementary Fig. 3). Finally, curved elongated areas, from 0.5 to 2 mm wide, with coalesced microaggregates were observed in BF3 and BF10 and can be interpreted as remnants of walls of galleries or cavities of several millimeters in diameter resulting from termite activity (Fig. 1d, Supplementary Fig. 4). Such galleries or cavities with infilling of loosely or closely-packed microaggregates were earlier described in optical microscopy as resulting from the activity of termites or ants^23,43,45,46^. Thus, half of the ferralic B horizons studied showed the presence of remnants of galleries or cavities resulting from the activity of termites or ants.

### Distribution of mineralogical markers of the activity of social insects

**Figure 2:**
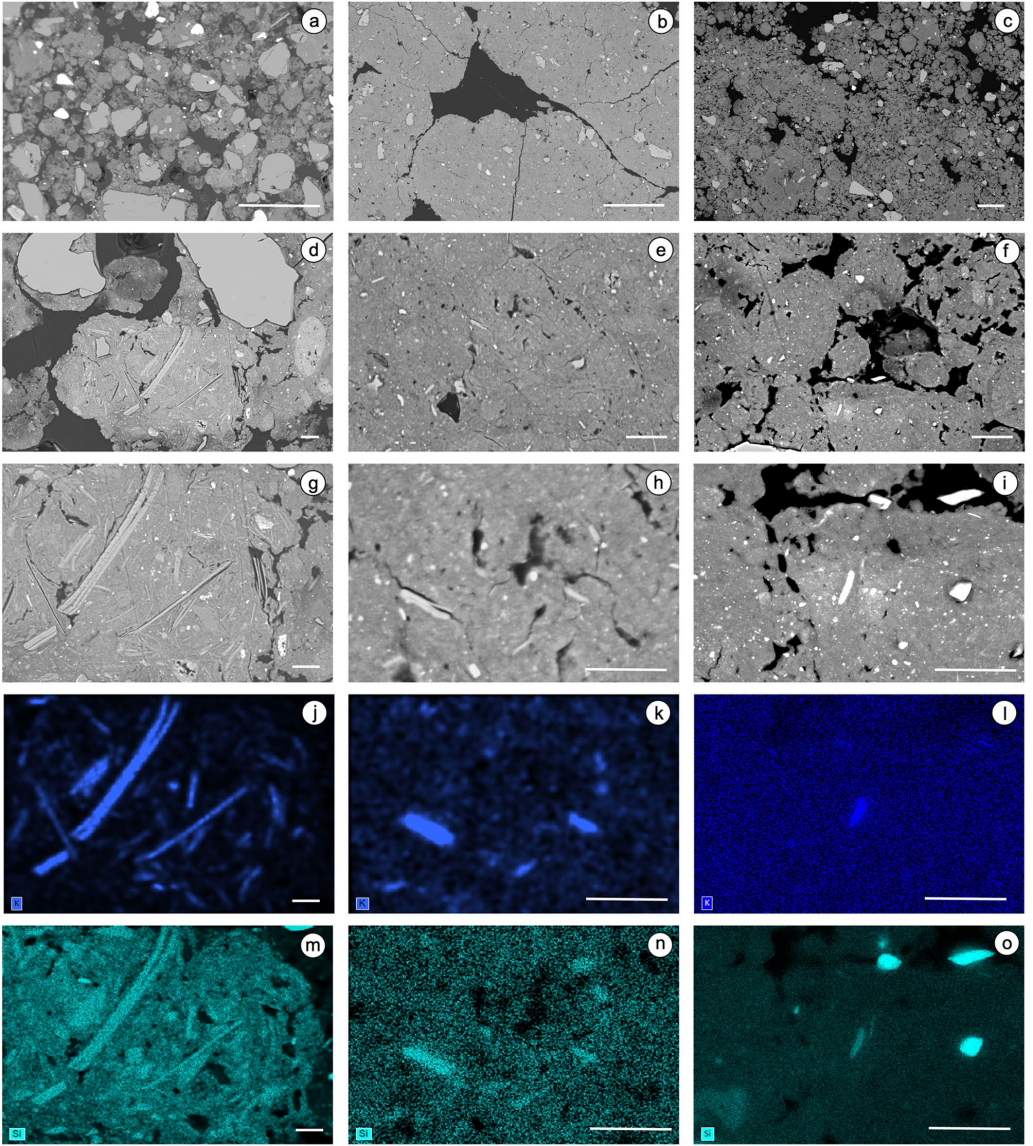
Observation of elongated clay particles with varying sizes and concentration in backscattered electron scanning images (BESI) and chemical analysis performed by using energy dispersive spectrometry (EDS) in BF1 (**a**,**d**,**g**,**j**,**m**), BF9 (**b**,**e**,**h**,**k**,**n**) and BF10 (**c**,**f**,**i**,**l**,**o**). BESI at low magnification show the development of microstructure with highly to moderately separated microaggregates forming a strong microgranular structure in BF1 (a and c) and BF10 and of a moderate to weak microgranular structure with coalesced microaggregates. Potassium 2:1 phyllosilicates are easily recognizable on BESI at high magnification and the map of the K (**j**–**l**) and Si (**m**–**o**) distribution in the latter. Bar length: 400 μm (**a**–**c**), 20 μm (**d**–**f**), 10 μm (**g**–**o**).

We then looked for other evidence of the activity of social insects by studying the distribution of potassium 2:1 clay minerals which have been earlier discussed as being markers of termite activity^11,21^. Given the difficulty of distinguishing what results from the activity of termites from that of ants, we consider here that the presence of 2:1 minerals is related to the activity of these social insects present in the soil studied. Analysis of the cross-sections showed the presence of potassium 2:1 clay minerals in all the ferralic B horizons studied (Table 1). They were identified by coupling backscattered electron scanning images (BESI) and images of the concentration of K and of Si using energy dispersive X-ray spectroscopy (EDS) (Fig. 2). Many very large elongated particles 50 to 500 μm long corresponding to these potassium 2:1 clay minerals associated to numerous smaller elongated particles with similar chemical composition were observed in BF1 (Table 1). In the other ferralic B horizons, all the elongated particles corresponding to the potassium 2:1 clay minerals were less than 50 μm long, except in BF3 and BF10 where they were less than 20 μm (Table 1). Whatever their size and their number in the different size classes, these markers of the activity of termites or ants were present in highly to moderately separated subangular microaggregates forming the strong microgranular structure as well as in the coalesced subrounded microaggregates forming the weak microstructure corresponding to the remnants of walls of galleries or cavities (Figs. 1 and 2).

**Table 1:** Length and number of elongated particles with K_2_O content ranging from 0.5 to 12% which were observed on the backscattered electron scanning images (BESI) of the polished Sects. 4.9 cm^2^ in surface area (diameter of 2.5 cm) of the ferralic B horizon of the Ferralsols studied.

Ferralic B horizon	Length and number of the elongated particles			
	< 2 µm	2–20 µm	20–50 µm	50–500 µm
BF1	10–50	10–50	10–50	10–50
BF2	10–50	1–10	1–10	n.o
BF3	10–50	1–10	n.o	n.o
BF4	10–50	1–10	n.o	n.o
BF5	> 50	> 50	1–10	n.o
BF6	> 50	10–50	1–10	n.o
BF7	> 50	> 50	1–10	n.o
BF8	> 50	> 50	1–10	n.o
BF9	> 50	> 50	10–50	n.o
BF10	> 50	> 50	n.o	n.o

n.o., not observed.

### Chemical composition of the markers of the mineralogical markers

Our results show that whatever the type of microgranular structure, potassium 2:1 phyllosilicates were present in all the ferralic B horizons studied (Table 1)^11,21^. They were present in different proportions in the microaggregates with some variations in the chemical composition depending on the ferralic B horizon studied presumably because of variations in the mineralogical composition of the parent material (Table 2)^23,47,48^. The structural formula was computed for these 2:1 phyllosilicates on the basis of the structure of a 2:1 phyllosilicate and for punctual chemical analysis of elongated particles exhibiting a K_2_O content ranging from 7.0 to 12.4% of the mass of oxides, the latter K_2_O content corresponding to a theoretical muscovite^49^. Results showed similar averaged structural formulas with substitution of Si^4+^ by Al^3+^ in the tetrahedral sites with a mean number of Al^3+^ ranging from 0.91 (BF6) to 1.18 (BF4) per half unit cell and a mean number of K^+^ in the inter-layer space ranging from 0.60 (BF5) to 0.89 (BF10) per half unit cell for the ten ferralic B horizons studied (Table 2). Results also showed that the mean number of Fe^3+^ in the octahedral sites ranged from 0.15 (BF1) to 0.41 (BF10) with a high variability between the analyses in every ferralic horizon studied (Table 2, Supplementary Table). Some particles also showed the presence of Na^+^ in the inter-layer space with a mean number of Na^+^ per half-unit cell of 0.13 in BF1, 0.10 in BF9 and 0.08 in BF5 (Table 2). The averaged structural formulas computed showed that the mean number of octahedral cavities occupied per half-unit cell ranged from 2.07 (BF6 and F10) to 2.16 (BF3), whereas the number of octahedral cavities per half-unit cell occupied is 2.00 for a dioctahedral 2:1 phyllosilicate^49^. The small excess of octahedral cavities occupied could be related to the presence of hydroxy-Al in the inter-layer space (Table 2, Supplementary Table). Consequently, part of Al^3+^ could be located inside the inter-layer space and not exclusively in the tetrahedral and octahedral cavities as assumed for computation of the structural formulas. The small deficit of positive charges in the inter-layer space compared to the negative charges resulting from substitutions in the tetrahedral and octahedral cavities could also be related to the presence of hydroxy-Al in the inter-layer space, thus blocking exchangeable sites which were initially occupied by K^+^ or Na^+^ in the poorly weathered muscovite (Table 2)^49^. However, whatever the small variations of the averaged structural formulas recorded, they are all those of potassium dioctahedral 2:1 phyllosilicates similar to those identified as markers of soil-feeding termite activity. Those with the highest mean number of K^+^ per half-unit cell in the inter-layer space correspond to poorly weathered muscovite and those with the smallest mean number of K^+^ in the inter-layer space to hydroxy-Al interlayered vermiculites resulting from deeper weathering of muscovite^11,21,49^.

**Table 2:** Averaged structural formula computed for the elongated particles showing a K_2_O content greater than 7% in every ferralic B horizon of Ferralsol studied.

Ferralsol	n.p	n.a	$\left[{\mathrm{Si}}_{\mathrm{a}}^{4+}{\mathrm{Al}}_{\mathrm{b}}^{3+}\right]{\mathrm{O}}_{10}^{2-}\left[{\mathrm{Al}}_{\mathrm{c}}^{3+}{\mathrm{Fe}}_{\mathrm{d}}^{3+}{\mathrm{Mg}}_{\mathrm{e}}^{2+}{\mathrm{Ti}}_{\mathrm{f}}^{4+}\right]{\left(\mathrm{OH}\right)}_{2}^{-}{\mathrm{K}}_{\mathrm{g}}^{+}{\mathrm{Na}}_{\mathrm{h}}^{+}{\mathrm{Ca}}_{\mathrm{i}}^{2+}$									X	Y
			a	b	C	d	e	f	g	h	i		
BF1	5	23	3.08	0.92	1.75	0.15	0.14	0.04	0.64	0.13	0.01	2.08	0.78
BF2	6	22	2.95	1.05	1.74	0.22	0.11	0.03	0.78	0.02	0.01	2.10	0.81
BF3	4	15	2.86	1.14	1.78	0.24	0.10	0.04	0.70	0.04	0.01	2.15	0.74
BF4	3	9	2.82	1.18	1.65	0.34	0.12	0.03	0.81	0.02	< 0.01	2.14	0.84
BF5	2	8	3.02	0.98	1.66	0.29	0.14	0.04	0.60	0.08	0.01	2.13	0.69
BF6	6	21	3.09	0.91	1.72	0.18	0.15	0.02	0.73	0.05	< 0.01	2.08	0.79
BF7	4	12	3.04	0.96	1.68	0.28	0.10	0.03	0.75	0.02	0.01	2.08	0.78
BF8	9	32	3.00	1.00	1.72	0.25	0.08	0.03	0.78	0.04	0.01	2.07	0.84
BF9	2	5	3.06	0.94	1.79	0.19	0.10	0.02	0.63	0.10	< 0.01	2.09	0.73
BF10	6	20	2.99	1.01	1.47	0.41	0.15	0.04	0.89	0.01	0.01	2.07	0.91

n.p., number of particles analyzed; n.a., number of analyses; X, number of octahedral cavities occupied per half-unit cell (c + d + e + f); Y, sum of the charges of the cations in the inter-layer space.

### Progressive integration of saprolite material into the microaggregates and implications

**Figure 3:**
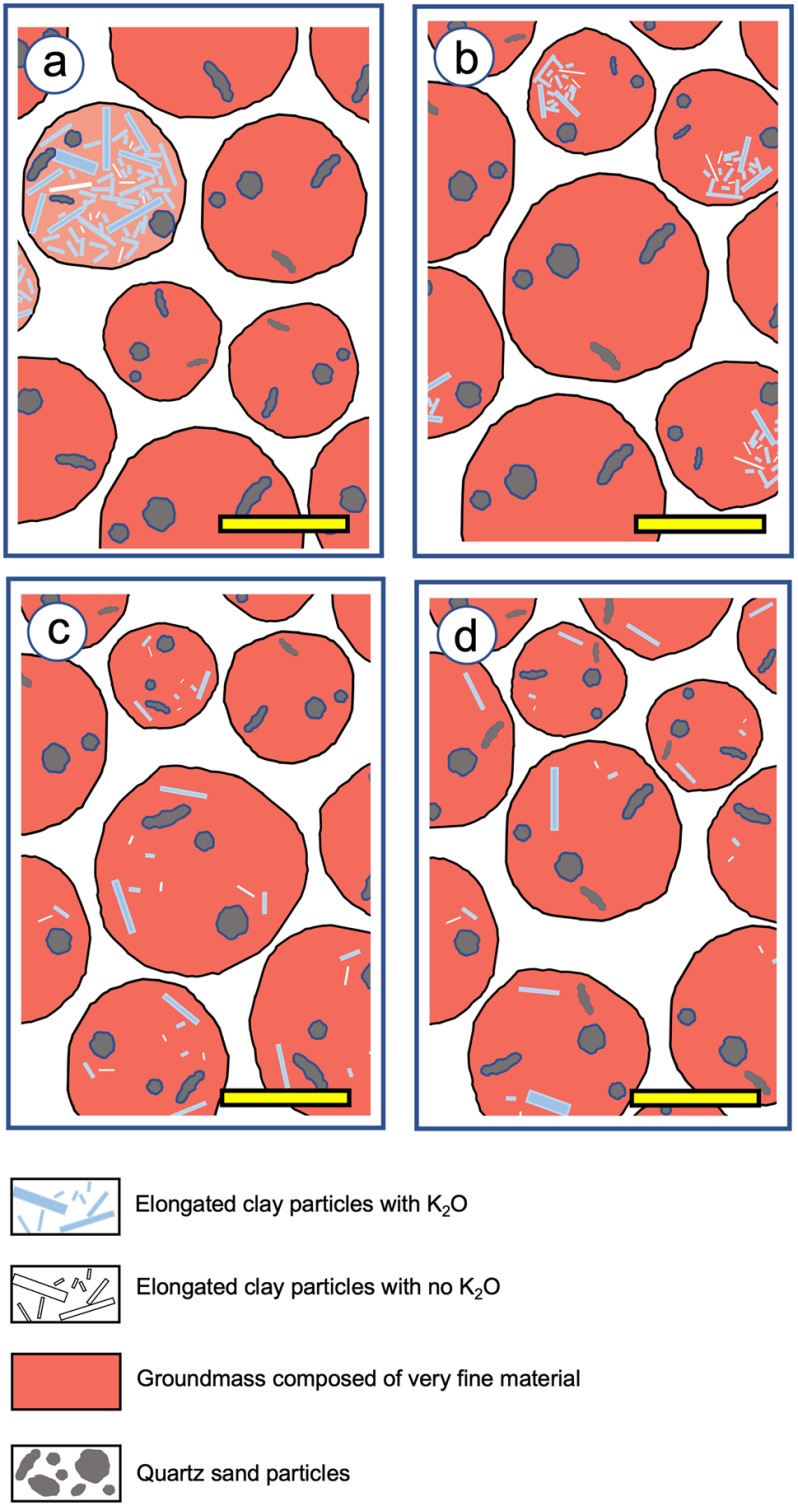
Schematic representation of the progressive integration from (**a**) to (**c**) of the material brought up by the activity of termites or ants from the saprolite. In (**a**), some microaggregates in the ferralic B horizon are directly inherited from the saprolite with a high content of minerals specific to the latter. Then, these allochthonous minerals are progressively incorporated in an increasing number of microaggregates thanks to the excavating and mixing activity of termites or ants (from **b** to **d**). Bar length: 500 μm.

The process responsible for the presence of these mineralogical markers of the activity of termites or ants starts with the uptake several meters deeper of small volumes of saprolite rich in poorly to highly weathered phyllitic minerals and their incorporation in ferralic B horizons. This was observed earlier in the BF6 studied here^21^ as well as in BF1 in this study (Fig. 2a). Although the motivation for such upward transport of small volumes of saprolite material by termites or ants is still highly debated^2,50–53^, galleries resulting from the excavating activity of termites were earlier observed in the field in the underlying saprolite and regolith^2^. They were indeed found at several tens of meters depth in South American, African and Australian regoliths^2,54,55^ and even during the seventies at a depth of about 70 m in a Brazilian regolith^56^. This excavating activity was interpreted as a clay mining activity to bring to the topsoil the clay material required to build stable structures able to maintain appropriate moisture conditions in the termite mount^2,51,55^. While this can be assumed for sandy soils^57^, it is not the case for the soils of this study which are for the most part very clayey^23^. As shown in several experimental studies, the need for termites to have access to nutrients such as K^+^ and Na^+^ in sufficient quantity while the subsoil is largely depleted of them could explain the upward transport of saprolite-rich clays with exchangeable K^+^ and Na^+^^50–52,58^. Whatever the motivations which lead the termites or ants to transport saprolite material upward, the result is an excavating activity that reorganizes the soil, resulting in gallery or cavity walls composed of subrounded, closely-packed microaggregates within a groundmass composed of sub-angular, loosely-packed microaggregates resulting from the long-term work of fragmentation, excavating and transport activity, thus mixing the material that resulted from geochemical evolution of the whole soil on a geological time scale^7–9^. Then, saprolite material is gradually incorporated and diluted into an increasing number of microaggregates as shown in Fig. 3, the allochthonous 2:1 phyllosilicates continuing, whatever their K_2_O content, to be weathered in contact with the soil solution.

Therefore, the physical fertility of the Ferralsols appears to be largely related to the activity of termites and ants activity which has taken place over the last hundreds of thousands of years, if not older because the deep tropical weathering occurred mainly during the Tertiary, and which has seen variations in climatic conditions and subsequently in soil fauna and vegetation^2,59–61^. Today it is difficult to assess the consequences of intensive agriculture on such highly microaggregated Ferralsols because this is a recent change in Brazil. If an evolution of the microgranular structure of Ferralsols, and consequently of their physical fertility, is in progress in Brazil following intensive cultivation, it is probably still barely perceptible, if at all. However, it is the ability of these soils to infiltrate water from intense tropical rains and thus to control runoff and the resulting soil erosion that are in question if the microgranular structure of Ferralsols deteriorates^19,20^. Consequently, detailed studies of the consequences of intense cultivation on the biodiversity of the termite and ants populations, particularly those responsible for the microgranular structure of Ferralsols, which were initially present under native vegetation, is highly indicated and needs to be encouraged.

## Methods

### The soils studied

The Ferralsols studied are located in the Brazilian Central Plateau where two main geomorphologic surfaces can be identified: the Late Tertiary South American Surface which corresponds to tablelands (usually 900 to 1200 m high) where gibbsitic-sesquioxidic Ferralsols are dominant and the Late Quaternary Velhas Surface (5 to 25 m below the South American Surface) which shows a moderate slope where kaolinitic-non-sesquioxidic Ferralsols are numerous^23,62,63^. The most representative climate of the Brazilian Central Plateau is Megathermic or Humid Tropical (Aw) with the savanna subtype^63^. It is characterized by maximum rains in summer and a dry winter (average temperature of the coldest month > 18 °C). The average annual rainfall ranges from 1500 to 2000 mm (Peel et al.^64^). Ten ferralic B horizons (BF1 to BF10) of Ferralsols (F1 to F10) corresponding to Ferralsols studied earlier (L1 to L10) were selected^23,47,48^. The location and main characteristics of these Ferralsols (Orthic Ferralsols: F2, F5 and F7; Rhodic Ferralsols: F1, F6, F8, F9 and F10; Xanthic Ferralsols: F3; Plinthic Ferralsols: F4) (WRB) which belong either to the South American Surface (BF2 to BF4) or to the Velhas Surface (BF1 and BF5 to BF6) can be found in these earlier studies^23,47^. They were all located under native forest vegetation at least 30 m away from any visible termite mount. They developed on a large range of parent materials (granulite: F1; sandy metharithimite: F2 and F3; quartzite: F4; clayey metharithimite: F5; metapelite: F6, F7 and F8; metapelite and limestone: F9; limestone: F10)^48,65^. Their ferralic B horizons were collected at a depth ranging from 0.85 m (BF4) to 1.70 m (BF6) and were highly clayey with a clay content ranging from 520 (BF1) to 780 g kg^−1^ (BF6)^48,65^. Their kaolinite content ranged from 196 (BF1) to 645 g kg^−1^ (BF9), their gibbsite content from 183 (BF9) to 625 g kg^−1^ (BF4), their hematite content from 0 (BF3 and BF4) to 205 g kg^−1^ (BF1) and their goethite content from 0 (BF10) to 178 g kg^−1^ BF4)^65^. More recent studies were conducted on BF2 and BF5^11^ and then on BF1, BF4 and BF6^21^. In similar soils in the Cerrado region, it was shown that most soil macroinvertebrates under native vegetation were termites taxons (76.0% of the total density in ind. m^−2^) when formicidae taxons were much less present (8.9% of the total density in ind. m^−2^)^66^. These results are consistent with those recorded earlier^38^. Moreover, several studies were dedicated to the inventory of families of termites and ants present in this region under native Cerrado vegetation^66,67^.

### Scanning electron microscopy

Undisturbed samples were collected, dried and then embedded in a polyester resin^68^. After polymerization and hardening, circular cross sections 2.5 cm in diameter were prepared and carbon coated for examination by scanning electron microscopy (SEM) using backscattered electron scanning images (BESI)^66^. Multiple observations at low magnifications (× 30 to × 40) were assembled to map the development of the microgranular structure visible on the cross Sects. 2.5 cm in diameter. Observations at higher magnifications (× 500 to × 5000) were used to identify particles of phyllosilicates in the groundmass of the microgranular aggregates^11,21^. The scanning electron microscope (SEM) used was a Merlin Compact Zeiss microscope (resolution of 0.8 nm at 15 kV and 1.6 nm at 1 kV; voltage ranging from 20 V to 30 kV; probe current ranging from 12 pA to 100 nA). It was equipped with a Gemini I column including a backscattered electron detector (BSD) with five quadrants for acquisition of the backscattered electron scanning images (BESI). Observations were performed at 15 kV accelerating voltage and at a working distance of 10 mm.

### Energy dispersive spectroscopy and calculation of the structural formulas

Chemical analyses were performed using energy dispersive X-ray spectroscopy (EDS) with a Quantax XFlash6 Bruker detector enabling a resolution of 129 eV. Analyses were performed also at 15 kV accelerating voltage. The SEM was operated with a resolution of 0.8 nm and a probe current of 1.6 nA. A count time of 100 s was used for punctual analyses. Total chemical composition was expressed on the basis that the sum of oxide mass equals 100 for determinations of SiO_2_, Al_2_O_3_, Fe_2_O_3_, MgO, CaO, K_2_O, and Na_2_O and TiO_2_^11,21^. Images of the concentration of K throughout the images were recorded with an acquisition time of 5 min. Chemical composition of the half unit cell was computed on the basis of the structural formula of a dioctahedral 2:1 phyllosilicate as structural model after location of the whole Al^3+^ first in the tetrahedral cavities to obtain the four cavities occupied by Si^4+^ and Al^3+^, then the remaining Al^3+^ in the octahedral cavities^11,21^. As for the whole Fe^3+^, Mg^2+^ and Ti^4+^, they were located in the octahedral cavities and the whole K^+^, Na^+^ and Ca^2+^ in the inter-layer space to equilibrate the negative charge of the layer which resulted from substitutions of Si^4+^ by Al^3+^ in the tetrahedral cavities and of Al^3+^ by Mg^2+^ and Ti^4+^ in the octahedral cavities.

## Supplementary Information

Supplementary Information 1.

Supplementary Information 2.

## Data Availability

The datasets used during the current study are available from the corresponding author on reasonable request.
