# Supplementary material for: Long-term activity of social insects responsible for the physical fertility of soils in the tropics
Source: Sci Rep. 2023 Jul 31;13:12337. doi: 10.1038/s41598-023-39654-w (PMC10390474; doi:10.1038/s41598-023-39654-w)
Supplement: Supplementary file 1 — Supplementary Information 1. [file 41598_2023_39654_MOESM1_ESM.docx]

Supplementary information

Long-term activity of social insects responsible

for the physical fertility of soils in the tropics

Ary Bruand^1*^, Adriana Reatto^2^, Michel Brossard^3^,

Pascal Jouquet^4^, Éder de Souza Martins^5^

^1^ Université d’Orléans, Institut des Sciences de la Terre d'Orléans (ISTO), UMR7327, UO–CNRS–BRGM, Observatoire des Sciences de l’Univers en région Centre (OSUC), Université d’Orléans, 1A Rue de la Férollerie, 45071 Orléans, Cedex 2, France.

^2^ Empresa Brasileira de Pesquisa Agropecuária (Embrapa), Secretaria de Pesquisa e Desenvolvimento, Parque Estação Biológica-PqEB s/n^0^, Brasília-DF, Brazil.

^3^ Institut de Recherche pour le Développement (IRD), Eco&Sols, UMR IRD–INRAE–CIRAD–Institut Agro–University of Montpellier, Montpellier, France.

^4^ Institut de Recherche pour le Développement (IRD), Institute of Ecology and Environmental Sciences of Paris (iEES Paris), UMR Sorbonne Université–UPEC–CNRS–IRD-INRAE, Paris, France.

^5^ Empresa Brasileira de Pesquisa Agropecuária (Embrapa Cerrados), Brasília-DF, Brasil.

^*^ Corresponding author: Ary Bruand ([Ary.Bruand@univ-orleans.fr](mailto:Ary.Bruand@univ-orleans.fr))


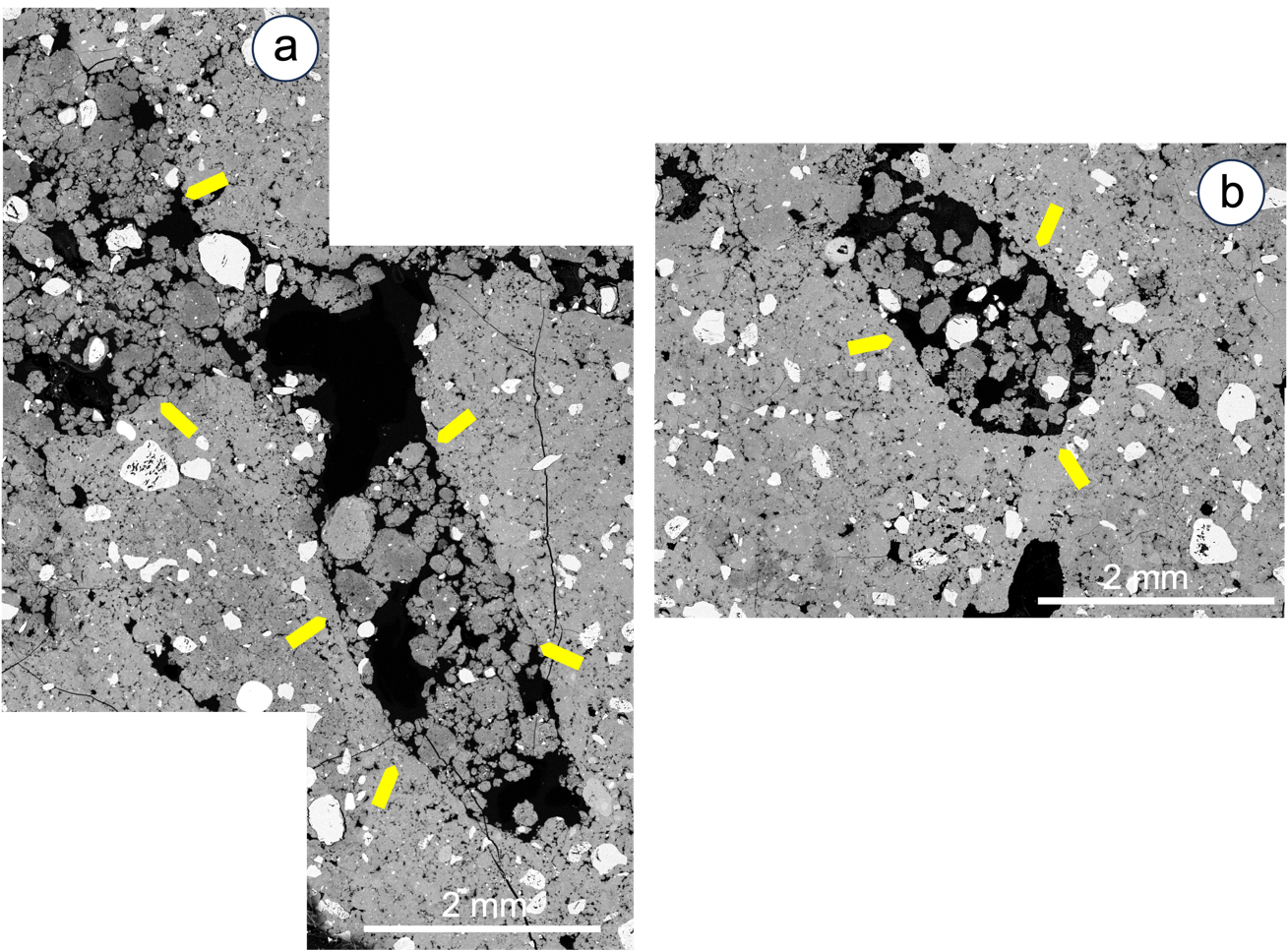


**Supplementary Fig. 1**| Detailed pictures of cross-sections of channels or cavities resulting (a and b) from termite or ant activity which are partially filled by microaggregates (BF2, yellow arrows). They were located within areas with a moderate to weak microgranular structure with coalesced subrounded microaggregates.


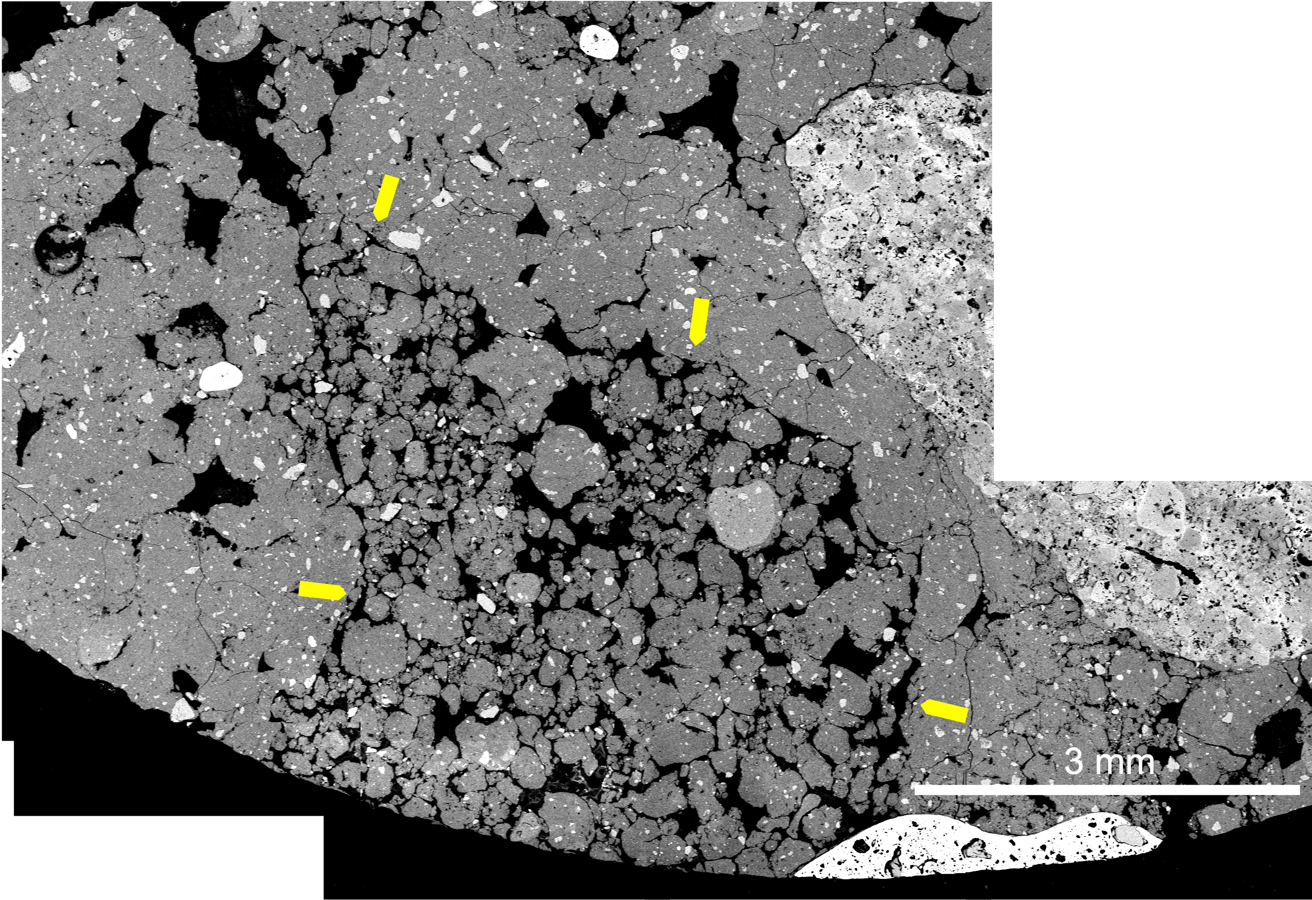


**Supplementary Fig. 2**| Detailed picture of the cross-section of a channel or a cavity resulting from termite or ant activity which is filled by microaggregates (BF7, yellow arrows). They were located within an area with a moderate to weak microgranular structure with coalesced subrounded microaggregates.


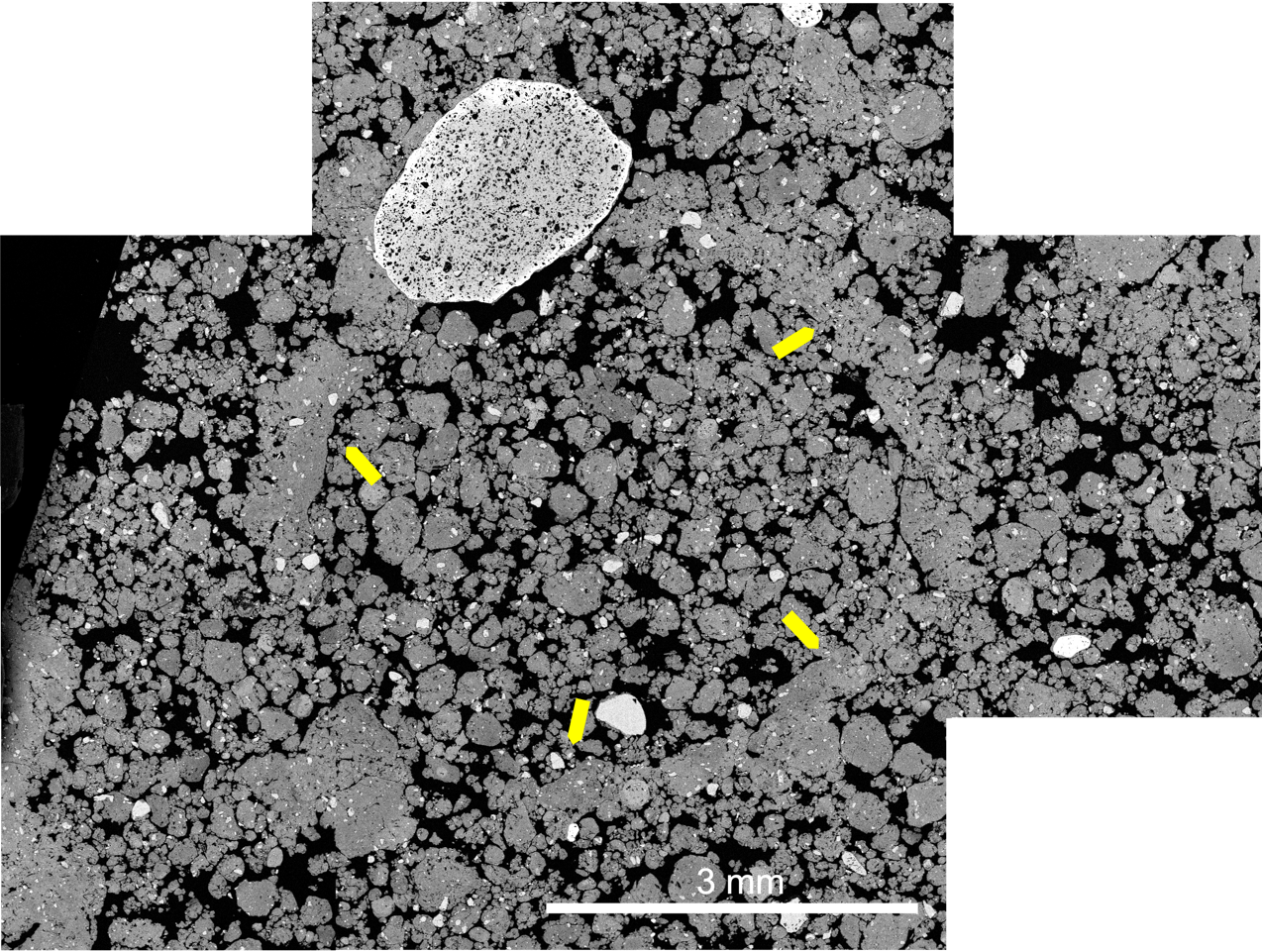


**Supplementary Fig. 3**| Detailed picture of the cross-section of a channel or a cavity resulting from termite activity as indicated by the walls which result from microaggregates glued together forming a close packing (BF9, yellow arrows). The channel or cavity was filled by microaggregates in a loose packing and located within an area with a strong microgranular structure with highly to moderately separated microaggregates.


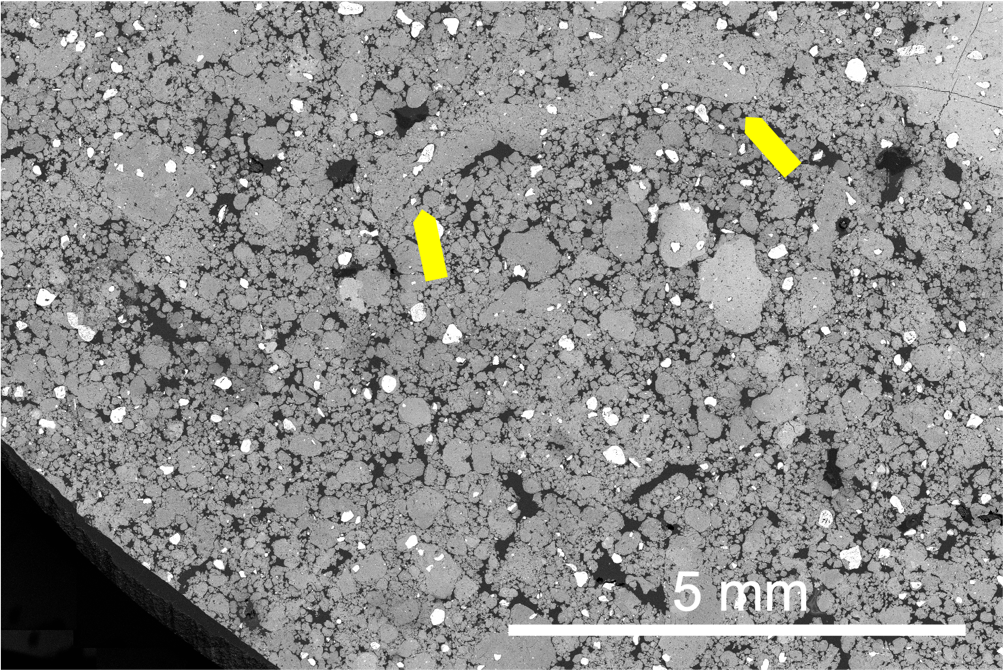


**Supplementary Fig. 4**| Detailed picture of the cross-section of a fragment of the wall of a channel or a cavity resulting from termite activity as indicated by the walls which result from microaggregates glued together forming a close packing (BF3, yellow arrows).

**Supplementary Table**| Averaged structural formula computed for the elongated particles showing a K_2_O content greater than 7% in every ferralic B horizon of Ferralsol studied. n.p.: number of particles analyzed; n.a.: number of analyzes; s.d.: standard deviation; min.: minimum value recorded; max.: maximum value recorded; X: number of octahedral cavities occupied by half-unit cell (c+d+e+f); Y: sum of the charges of the cations in the inter-layer space.

| Ferralsol | n.p. | n.a. | $\left[ \mathrm{Si}_{a}^{4+}\mathrm{Al}_{b}^{3+} \right]O_{10}^{2-}\left[ \mathrm{Al}_{c}^{3+}\mathrm{Fe}_{d}^{3+}\mathrm{Mg}_{e}^{2+}\mathrm{Ti}_{f}^{4+} \right]\left( \mathrm{OH} \right)_{2}^{-}K_{g}^{+}\mathrm{Na}_{h}^{+}\mathrm{Ca}_{i}^{2+}$ | | | | | | | | | | X | Y |
| --- | --- | --- | --- | --- | --- | --- | --- | --- | --- | --- | --- | --- | --- | --- |
|  |  |  |  | a | b | c | d | e | f | g | h | i |  |  |
| BF1 | 5 | 24 | mean | 3.08 | 0.92 | 1.75 | 0.15 | 0.14 | 0.04 | 0.64 | 0.13 | 0.01 | 2.08 | 0.78 |
|  |  |  | s.d. | 0.02 | 0.02 | 0.08 | 0.06 | 0.03 | 0.01 | 0.04 | 0.04 | <0.01 | 0.02 | 0.04 |
|  |  |  | min. | 3.02 | 0.88 | 1.55 | 0.09 | 0.09 | 0.03 | 0.58 | 0.05 | <0.01 | 2.05 | 0.73 |
|  |  |  | max. | 3.12 | 0.98 | 1.84 | 0.32 | 0.20 | 0.06 | 0.71 | 0.23 | 0.01 | 2.11 | 0.87 |
| BF2 | 6 | 22 | mean | 2.95 | 1.05 | 1.74 | 0.22 | 0.11 | 0.03 | 0.78 | 0.02 | 0.01 | 2.10 | 0.81 |
|  |  |  | s.d. | 0.15 | 0.15 | 0.12 | 0.08 | 0.12 | 0.02 | 0.16 | 0.02 | 0.01 | 0.10 | 0.16 |
|  |  |  | min. | 2.58 | 0.93 | 1.52 | 0.09 | 0.01 | 0.01 | 0.55 | <0.01 | 0.01 | 1.98 | 0.58 |
|  |  |  | max. | 3.07 | 1.42 | 1.89 | 0.37 | 0.44 | 0.06 | 1.06 | 0.08 | 0.01 | 2.28 | 1.08 |
| BF3 | 4 | 15 | mean | 2.86 | 1.14 | 1.78 | 0.24 | 0.10 | 0.04 | 0.70 | 0.04 | 0.01 | 2.15 | 0.74 |
|  |  |  | s.d. | 0.13 | 0.13 | 0.11 | 0.12 | 0.05 | 0.01 | 0.14 | 0.03 | <0.01 | 0.09 | 0.16 |
|  |  |  | min. | 2.62 | 0.99 | 1.52 | 0.15 | 0.05 | 0.02 | 0.42 | 0.01 | <0.01 | 1.99 | 0.45 |
|  |  |  | max. | 3.01 | 1.38 | 1.94 | 0.59 | 0.19 | 0.05 | 0.99 | 0.08 | 0.01 | 2.31 | 1.08 |
| BF4 | 3 | 9 | mean | 2.82 | 1.18 | 1.65 | 0.34 | 0.12 | 0.03 | 0.81 | 0.02 | <0.01 | 2.14 | 0.84 |
|  |  |  | s.d. | 0.20 | 0.20 | 0.07 | 0.15 | 0.04 | 0.01 | 0.15 | 0.01 | <0.01 | 0.27 | 0.17 |
|  |  |  | min. | 2.46 | 0.93 | 1.53 | 0.18 | 0.04 | 0.02 | 0.63 | <0.01 | <0.01 | 1.78 | 0.63 |
|  |  |  | max. | 3.07 | 1.54 | 1.77 | 0.67 | 0.16 | 0.05 | 1.05 | 0.07 | 0.01 | 2.65 | 1.14 |
| BF5 | 2 | 8 | mean | 3.02 | 0.98 | 1.66 | 0.29 | 0.14 | 0.04 | 0.60 | 0.08 | 0.01 | 2.13 | 0.69 |
|  |  |  | s.d. | 0.06 | 0.06 | 0.15 | 0.15 | 0.08 | 0.02 | 0.17 | 0.07 | <0.01 | 0.08 | 0.20 |
|  |  |  | min. | 2.95 | 0.88 | 1.35 | 0.18 | 0.06 | 0.02 | 0.30 | <0.01 | <0.01 | 2.07 | 0.35 |
|  |  |  | max. | 3.12 | 1.05 | 1.81 | 0.63 | 0.25 | 0.08 | 0.79 | 0.17 | 0.01 | 2.27 | 0.85 |
| BF6 | 6 | 21 | mean | 3.09 | 0.91 | 1.72 | 0.18 | 0.15 | 0.02 | 0.73 | 0.05 | <0.01 | 2.08 | 0.79 |
|  |  |  | s.d. | 0.17 | 0.17 | 0.11 | 0.08 | 0.13 | 0.01 | 0.08 | 0.05 | 0.01 | 0.05 | 0.08 |
|  |  |  | min. | 2.86 | 0.53 | 1.48 | 0.06 | 0.05 | 0.01 | 0.58 | <0.01 | <0.01 | 2.01 | 0.63 |
|  |  |  | max. | 3.47 | 1.14 | 1.85 | 0.38 | 0.47 | 0.05 | 0.92 | 0.16 | 0.02 | 2.18 | 0.93 |
| BF7 | 4 | 12 | mean | 3.04 | 0.96 | 1.68 | 0.28 | 0.10 | 0.03 | 0.75 | 0.02 | 0.01 | 2.08 | 0.78 |
|  |  |  | s.d. | 0.10 | 0.10 | 0.11 | 0.09 | 0.06 | 0.02 | 0.10 | 0.02 | 0.01 | 0.05 | 0.09 |
|  |  |  | min. | 2.89 | 0.79 | 1.52 | 0.13 | 0.02 | 0.00 | 0.58 | <0.01 | <0.01 | 2.02 | 0.59 |
|  |  |  | max. | 3.21 | 1.11 | 1.82 | 0.39 | 0.23 | 0.06 | 0.87 | 0.04 | 0.02 | 2.16 | 0.92 |
| BF8 | 9 | 32 | mean | 3.00 | 1.00 | 1.72 | 0.25 | 0.08 | 0.03 | 0.78 | 0.04 | 0.01 | 2.07 | 0.84 |
|  |  |  | s.d. | 0.08 | 0.08 | 0.17 | 0.18 | 0.06 | 0.01 | 0.10 | 0.02 | 0.01 | 0.05 | 0.11 |
|  |  |  | min. | 2.74 | 0.87 | 1.19 | 0.08 | 0.04 | 0.00 | 0.60 | 0.02 | <0.01 | 2.00 | 0.63 |
|  |  |  | max. | 3.13 | 1.26 | 1.90 | 0.88 | 0.24 | 0.04 | 1.06 | 0.08 | 0.02 | 2.23 | 1.11 |
| BF9 | 2 | 5 | mean | 3.06 | 0.94 | 1.79 | 0.19 | 0.10 | 0.02 | 0.63 | 0.10 | <0.01 | 2.09 | 0.73 |
|  |  |  | s.d. | 0.08 | 0.08 | 0.18 | 0.14 | 0.08 | 0.02 | 0.04 | 0.08 | <0.01 | 0.05 | 0.11 |
|  |  |  | min. | 3.00 | 0.80 | 1.49 | 0.09 | 0.03 | 0.00 | 0.59 | 0.01 | <0.01 | 2.05 | 0.61 |
|  |  |  | max. | 3.20 | 1.00 | 1.92 | 0.42 | 0.20 | 0.06 | 0.69 | 0.16 | 0.01 | 2.18 | 0.87 |
| BF10 | 6 | 20 | mean | 2.99 | 1.01 | 1.47 | 0.41 | 0.15 | 0.04 | 0.89 | 0.01 | 0.01 | 2.07 | 0.91 |
|  |  |  | s.d. | 0.13 | 0.13 | 0.19 | 0.14 | 0.06 | 0.01 | 0.16 | 0.01 | <0.01 | 0.09 | 0.16 |
|  |  |  | min. | 2.73 | 0.83 | 1.19 | 0.16 | 0.07 | 0.01 | 0.59 | <0.01 | <0.01 | 1.96 | 0.60 |
|  |  |  | max. | 3.17 | 1.27 | 1.77 | 0.61 | 0.23 | 0.07 | 1.20 | 0.04 | 0.02 | 2.27 | 1.24 |
